# Primary Stability Recognition of the Newly Designed Cementless Femoral Stem Using Digital Signal Processing

**DOI:** 10.1155/2014/478248

**Published:** 2014-04-01

**Authors:** Mohd Yusof Baharuddin, Sh-Hussain Salleh, Mahyar Hamedi, Ahmad Hafiz Zulkifly, Muhammad Hisyam Lee, Alias Mohd Noor, Arief Ruhullah A. Harris, Norazman Abdul Majid

**Affiliations:** ^1^Department of Biomedical Engineering, Faculty of Engineering, University of Malaya, 50603 Lembah Pantai, Kuala Lumpur, Malaysia; ^2^Centre for Biomedical Engineering Transportation Research Alliance, Universiti Teknologi Malaysia, 81310 Skudai, Johor, Malaysia; ^3^Department of Orthopaedic, Traumatology & Rehabilitation, Kuliyyah of Medicine, International Islamic University Malaysia, 25200 Kuantan, Pahang, Malaysia; ^4^Department of Mathematical Sciences, Faculty of Science, Universiti Teknologi Malaysia, 81310 Skudai, Johor, Malaysia

## Abstract

Stress shielding and micromotion are two major issues which determine the success of newly designed cementless femoral stems. The correlation of experimental validation with finite element analysis (FEA) is commonly used to evaluate the stress distribution and fixation stability of the stem within the femoral canal. This paper focused on the applications of feature extraction and pattern recognition using support vector machine (SVM) to determine the primary stability of the implant. We measured strain with triaxial rosette at the metaphyseal region and micromotion with linear variable direct transducer proximally and distally using composite femora. The root mean squares technique is used to feed the classifier which provides maximum likelihood estimation of amplitude, and radial basis function is used as the kernel parameter which mapped the datasets into separable hyperplanes. The results showed 100% pattern recognition accuracy using SVM for both strain and micromotion. This indicates that DSP could be applied in determining the femoral stem primary stability with high pattern recognition accuracy in biomechanical testing.

## 1. Introduction

Total hip arthroplasty (THA) has been the most successful surgery in the orthopaedic field in the 20th century. Common issues which arise concerning the femoral stem include stress shielding in the proximal calcar and micromotion within the femoral canal [1–4]. Stiffer femoral stems induce stress shielding and bone resorption due to lack of mechanical response in surrounding bone [2, 3]. This phenomenon which commonly occurs in proximal calcar will complicate the revision surgery either while removing the old stem or while providing primary stability for a new stem because of the severe bone resorption at that region [5]. In addition, the interface micromotion between the femoral stem and the medullary canal should be around 40 *μ*m to promote primary bone ingrowth and less than 150 *μ*m to prevent fibrous tissue formation [6, 7]. This is essential for cementless femoral stems which depend solely on the implant—bone interface for osseointegration and primary fixation stability. Stress distribution and micromotion are generally validated in vitro using human cadaveric bones. Still, the availability of cadaveric bone is restricted and its preparation is cumbersome. The use of composite femur is a solution which mimics the mechanical properties of actual human femora [8]. In addition, this eradicates geometrical and mechanical differences between cadaveric femoras [2].

Finite element analysis (FEA) has become an important tool for researchers to predict the results of the newly designed implant [9]. Pettersen et al. [1, 2] affirmed the correlation between subject specific human cadaveric femur and finite element analysis which looks upon the stress shielding and micromotion around cementless femoral stems. In addition, Dopico-González et al. [10] investigated a probabilistic finite element analysis of cementless femoral stems which emphasized femora anatomical features and geometrical stem design which demonstrated good agreement with the in vitro study. In this present study, we would like to utilize our knowledge of digital signal processing on strain and micromotion for pattern recognition. As far as the authors are aware, there is no documented study regarding feature extraction and pattern recognition involving either micromotion or strain distribution for primary stability of the cementless femoral stem involving a support vector machine as a classifier. Only few studies applied DSP while studying femoral stem loosening [11–13] and stress impact [14]. The conventional diagnostic investigation for loosening after THA generally uses imaging modalities such as plain radiograph, arthrography, scintigraphy, and fluorodeoxyglucose-positron emission tomography (FDG-PET) [11]. As more than one million THA are performed each year, better methods using sensors were introduced to reduce costs and improve diagnostic performances for THA loosening. The characterization of the femoral stem and bone is completed using electrical (conductivity), mechanical (strain, micromotion, and stiffness), acoustic (audible sound and ultrasound), and biological (pH and temperature) properties [11]. Li et al. [12] found that vibration analysis using frequency (fast Fourier transform) could be implemented to diagnose late loosening but it performed poorly when used to diagnose early loosening. Pastrav et al. [13] assessed the in vivo vibration analysis based end point during femoral stem implantation using a frequency response function that offered reliable information, stability, and lessen intraoperative fractures. Gueiral and Nogueira [14] studied the impact of peak stress on THA by employing acoustic emissions, normally used for detection, location, and classification of cracks in the femoral canal. The objectives of this study were (1) to validate the newly designed femoral stem from experimental and finite element analysis and (2) to propose a new method using support vector machine in feature extraction and pattern recognition of the femoral stem primary stability.

## 2. Materials and Methods

### 2.1. Experimental Protocol

**Figure 1:**
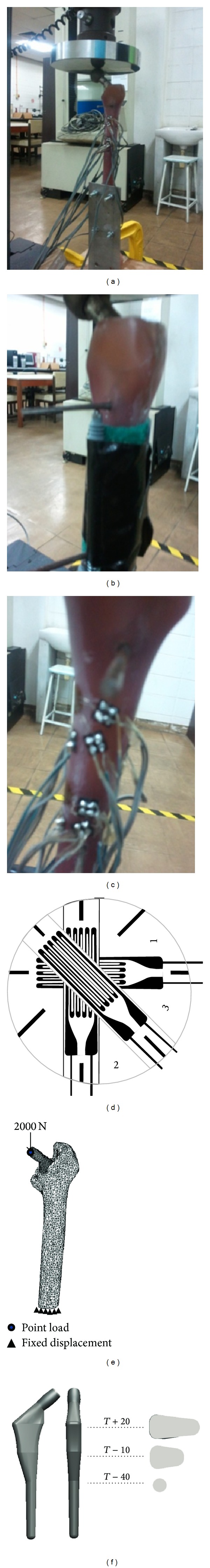
Experimental validation using composite femur (a) loading condition, (b) micromotion, (c) strain distribution, (d) triaxial rosette orientations, (e) finite element analysis, and (e) newly designed femoral stem.

The experiment was performed to validate the finite element analysis towards stress distribution and micromotion as shown in Figure 1. We used small left fourth generation composite femur (Sawbones, Pacific Research Laboratories Inc., Vashon Island, WA, USA) which mimicked actual human femora in accordance with Asian hip morphology. The composite femur had a 9.5 mm isthmus diameter with a collodiaphyseal angle of 130°. The femur neck was resected by an experienced orthopaedic surgeon before being implanted with our newly designed femoral stem within the medullary canal. This metaphyseal loading mediolaterally flared femoral stem was designed tailored to Asian femur anatomy [15, 16]. The femur was loaded at the center of the femoral stem ball using an advanced material testing system machine (Instron 5565, Norwood, MA, USA) at the rate of 1 kN/min and constrained distally using a custom designed jig positioned at the base of the machine. The cyclic axial loading was set from 0 to 2000 N using 5 kN load cell for 50 cycles. The jig aid aligned the vertical loading with the femur mechanical axis and was tilted 12° in valgus, mimicking the actual femur orientation. The pretest was done using a similar set-up before experimental validation to stabilize the implant within the femoral canal.

#### 2.1.1. Micromotion Measurement

Micromotion was measured using two linear variable displacement transducers (LVDT Model DP/2/S, Orbit3 Digital Probe, Solartron Metrology, West Sussex, UK) proximally and distally as shown in Figure 1(b). These sensors processed data up to 3906 readings per second, with an accuracy of 0.1 *μ*m, resolution of 1 *μ*m, and a measurement range of 2 mm. The calibration showed peak-to-peak error of 0.13–0.16 *μ*m while under 1014 mbars pressure. Four mm diameter holes were drilled 10 mm below the osteotomy level for the proximal region and 10 mm above the femoral stem tip for the distal region. The steel pins were glued into the femoral stem holes which were drilled prior to implantation to prevent a stem—medullary canal mismatch. The sensors were fixed firmly at the extra cortical femora with the spring tip touching the steel pin. Micromotion was measured by the LVDT once the axial cyclic loading from Instron machine was exerted upon the femoral stem through a digital network (Orbit3 Digital Network V3.0, Solartron Metrology, West Sussex, UK) connected to the sensors. Elastic micromotion was computed from the difference between the peak and trough for each cycle.

#### 2.1.2. Strain Measurement

Strain distribution was measured using four triaxial rosettes (UFRA-5-350-17, Tokyo Sokki Kenkyujo Co. Ltd, Tokyo, Japan) medially and laterally at the metaphyseal region as shown in Figure 1(c). This stainless steel (SUS 304) gauge had resistance of 350 ± 1.0 Ω and factor of 2.13 ± 1.0%. In addition, this 5 mm length gauge had three grid orientations 0° (*ϵ*_1_), 45° (*ϵ*_2_), and 90° (*ϵ*_3_) as illustrated in Figure 1(d). Several steps were taken to bind the triaxial rosette optimally to the composite femur.

1. The position of the strain gauge to the femur surface was first determined, proximally medial and lateral calcar.
2. The femur surface was prepared by removing any grease or dirt with a solvent (Freon TF). A region larger than the bonding area was wet abraded using silicon carbide paper (220–320 grit size) with a conditioner (M-Prep Conditioner A) and dried using gauze sponge.
3. The femur surface was then finely cleansed with a small amount of acetone (M-Prep Neutralizer 5) using a cotton tipped applicator to prevent contamination.
4. The strain gauge was carefully removed from the acetate envelope and tape mastic (M-M Number PCT-2 cellophane tape) was placed over the gauge and its lead to ease the realignment process. The adhesive (M-Bond 200) was then swabbed uniformly at the back of the strain gauge base.
5. The strain gauge was realigned and promptly applied to the femur surface and pressed down using thumb with tape mastic (M-M Number PCT-2 cellophane tape) over it for approximately one minute to complete the curing process.
6. The tape was gently removed and the gauge leads were raised using a pair of tweezers. A terminal foil shape connector (TF-2S) was placed near the gauge (3–5 mm) to alleviate the wiring process. The gauge leads were soldered slightly taut to the connecting terminal to avoid excessive tension during strain measurement. The extension lead wire was soldered to the terminal wire at the opposite side of the connecting terminal. The strain gauge was then protected with polyurethane protective layer (PU120). The terminal wires which connected to the strain gauge were finally connected to a multichannel data logger (TDS-630, Tokyo Sokki Kenkyujo Co. Ltd, Tokyo, Japan). The equivalent von Misses stress was computed using the strain data acquired.

### 2.2. Finite Element Analysis

The femoral stem was designed using computer aided design (CAD) software (SolidWorks 2009 SP2.1, Dassault System, Massachusetts, USA) in accordance with local anatomical femoral features [17–19]. The osteotomy level was set to 20 mm above the center of the lesser trochanter. The stem was subsequently aligned within the medullary canal to simulate hip arthroplasty and the stem neck was positioned to mimic the experimental stem orientation. The stem and “virtual surgery femora” were then imported to finite element software (Marc Mentat, MSC Software, Santa Ana, CA) in stereo lithographic format and then converted into solid linear first order tetrahedral elements. A mesh convergence study was performed on the femoral stem to ensure that the results were independent of the mesh density. An average of 13 200 elements with 4 200 nodes was found to be optimal for the cementless femoral stem, and the “virtual surgery femora” consisted of 7 900 nodes and 41 900 elements. The material properties of the cementless femoral stem were described as 316 L stainless steel with Young's Modulus of 200 GPa with a Poisson's ratio of 0.3 [20]. In addition, the femur was assumed to be isotropic and linear elastic, with bone properties determined according to the CT datasets grey level values using the correlation proposed by Carte and Hayes [21]. The cancellous and cortical bones were assumed to be at different ends of a continuum spectrum. The finite element model was completely restrained distally and loaded at the center of the femoral stem head with 2 kN as showed in Figure 1. A deformable to deformable contact was created between stem and femur with a friction coefficient of 0.4. The micromotion algorithm subroutine used in this study was written using Compaq Visual Fortran software (Compaq Computer Corporation) to compute micromotion in finite element software. The result focused on the equivalent von Mises stress and micromotion.

### 2.3. Digital Signal Processing

Root mean square (RMS) technique was used to feed the classifiers because the RMS provided the maximum likelihood estimation of amplitude in a constant force when a signal was modeled as a Gaussian random process. The micromotion signals for each channel (proximal and distal) were divided into three classes: high peak, transition, and stabilized. On the other hand, strain signal for each channel (*ɛ*_1_, *ɛ*_2_, and *ɛ*_3_) was divided into four classes (A, B, C, and D). The RMS was excerpted after every 500 seconds of raw signal and subsequently fed into classifier as shown in (1), where *x*_*n*_ are the signals from all datasets and *N* is the length of *x*_*n*_. Consider

(1) $$\begin{array}{c} \text{RMS}=\;\frac{1}{N}\overset{N}{\underset{n=1}{\sum }}x_{n}^{2}. \end{array}$$

In this present study, multiclass support vector machine (SVM) is used to classify the 3 classes of LVDT and 4 classes of triaxial rosette under consideration which required a classifier as shown in (2), where *k* is the number of classes which approximate the most suitable class from the datasets. Consider

(2) $$\begin{array}{c} f:R^{N}⟶\left\{1,\ldots ,k\right\},\\ \left(x_{1},y_{x1}\right),\ldots ,\left(x_{n},y_{n1}\right)\in R^{N}\times \left\{1,\ldots ,k\right\}. \end{array}$$

SVM is commonly used as a binary classifier to classify two groups of data. However, an increment to the datasets and classes required an optimal nonlinear classification with SVM which could solve the classification problems by mapping the original data into a “feature space”. The kernel function *φ*(·) was applied to the map training vector *x*_*i*_ into a higher dimensional space, which belonged to the dot product space as shown in

(3) $$\begin{array}{c} k\left(x_{i},x_{j}\right)=\;\left(\varphi \left(x_{i}\right)\cdot \varphi \left(x_{j}\right)\right). \end{array}$$

We applied the radial basis function (RBF) as the kernel types in accordance with our datasets structure, where *γ* > 0 is the kernel parameter as shown in

(4) $$\begin{array}{c} k\left(x_{i},x_{j}\right)=\;e^{-\gamma {\left|x_{1}-x_{j}\right|}^{2}}. \end{array}$$

Subsequently, these datasets were mapped into the linearly separable space, and hyperplanes divided them into two labeled classes. The hyper plane was the best option to separate the data as it yields the maximum margin of separation between the classes. One-against-all and one-against-one were two techniques used in multiclass SVM classification. In this study, one-against-all method was used to classify the data because this technique was easy to apply, required less computational time, and produced accurate results. Training one-against-all is an essential requisite of the *k* binary SVMs training. In addition, estimation for the probability of the output of a pairwise classifier between classes *i* and *j* is defined by *r*_*ij*_ as shown in

(5) $$\begin{array}{c} r_{ij}\approx p\;\left(y=i\;|\;y=\;\left\{i,j\right\},x\right),\;\;r_{ij}+r_{ji}=1 \end{array}$$

and *p*_*i*_ is the probability of the *i*th class. The class probability *p* = (*p*_1_,…, *p*_*k*_) can be derived by (6)

(6) $$\begin{array}{c} \min\overset{k}{\underset{i=1}{\sum }}\;\overset{}{\underset{j:j\neq 1}{\sum }}{\left(r_{ij}p_{j}-r_{ji}p_{i}\right)}^{2},\;\;\overset{k}{\underset{i=1}{\sum }}p_{i}=1P_{i}\geq 0. \end{array}$$

The SVM parameters were adjusted, and three- (micromotion) or four- (strain) fold random cross validation was employed for assessment purposes. As mentioned above, RBF was our kernel type (*γ* = 1/*k*) where *k* was the number of attributes in the input data and *C* = 1 was the cost of SVM. The active features were randomly permutated preceding training to facilitate classifier training. Subsequently, 70% of the data were fed to classifiers for training and 30% for testing in SVM.

### 2.4. Statistical Analysis

The time domain features for both interface micromotion and strain distribution were statistically analyzed with SAS 4.3 software (SAS Institute Inc., Cary, NC, USA). For micromotion, two cases were studied which involved the comparison between channels (proximal and distal) and the comparison between classes (high peak, transition, and stabilized) in each channel. On the other hand, three cases were studied in strain distribution which consisted of the comparison between channels (*ɛ*_1_, *ɛ*_2_, and *ɛ*_3_) in each class (A, B, C, and D), comparison between classes, and comparison between middle (AB) and lateral (CD) classes. Normality assumption for each group of data was verified using Kolmogorov-Smirnov method. Folded *F* method will be used to examine the equality of data variance if the data was normally distributed. The probability was then checked using *t*-test either by Pooled method or Satterthwaite method, according to the equality of the variance. If the data was not normally distributed, nonparametric one-way variance (ANOVA) was adopted using Wilcoxon scores. The value for probability (Pr > *F*) must be less than 0.05 to verify that the data were statistically significant for variance analysis. Subsequently, multiple comparisons were performed using Tukey's studentized range test using the least square mean for effect feature (Pr > |*t*|) which must be less than 0.05 to demonstrate that the comparison was statistically significant.

## 3. Results

**Figure 2:**
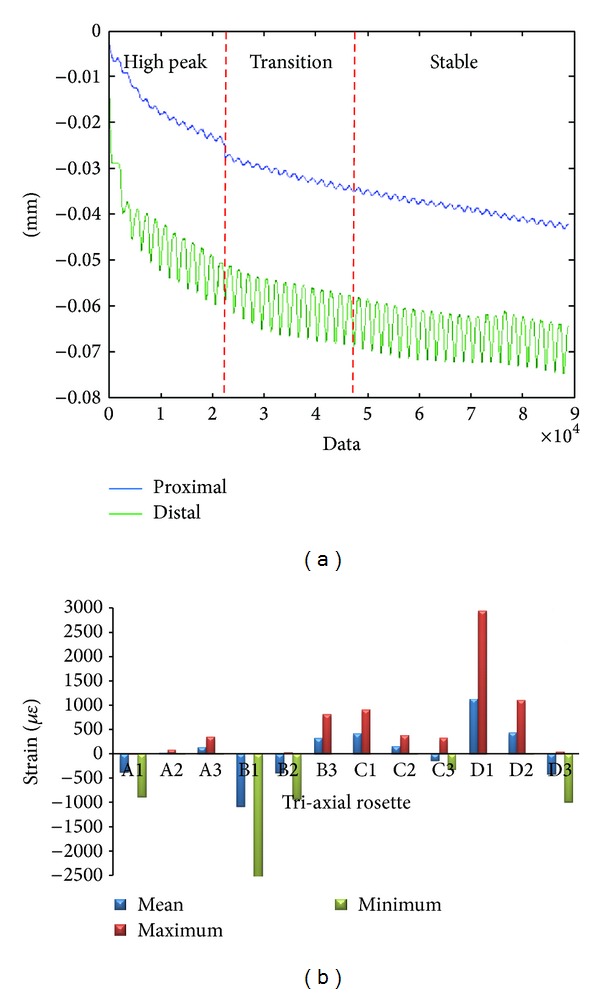
Raw data for (a) micromotion and (b) strain gauge.

**Figure 3:**
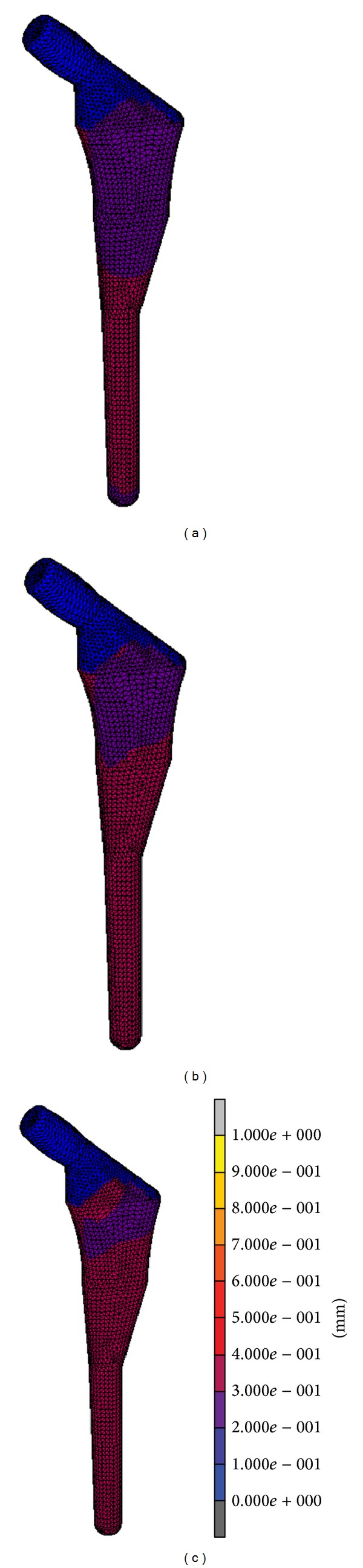
Finite element analysis for micromotion (a) high peak, (b) transition, and (c) stable phase.

**Figure 4:**
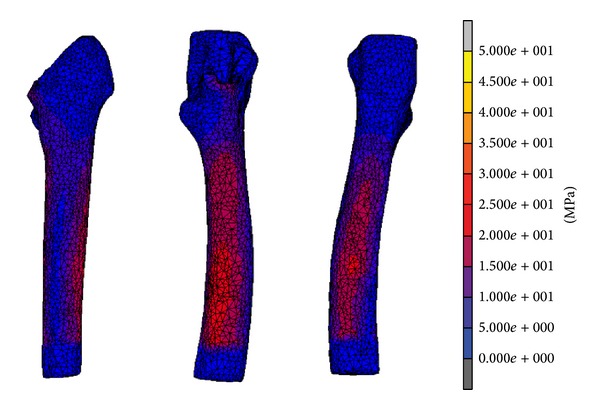
Finite element analysis for equivalent von Mises stress after stable phase.

The raw data for both micromotion and strain distribution are illustrated in Figure 2. For the micromotion, we divided the signal into three regions: high peak (initial phase), transition (middle phase), and stable (final phase). The signals decreased exponentially for both proximal and distal region. The raw signal for proximal region started with 4 *μ*m before stabilizing at *≈*40 *μ*m, while at distal region started with 18 *μ*m before stabilizing at *≈*70 *μ*m. The elastic micromotion was computed which showed proximal region stabilized at 1.5–2.0 *μ*m and distal region stabilized at 10–12 *μ*m. The strain signal is measured from four different locations around metaphyseal region: proximal medical calcar (A), distal medical calcar (B), proximal lateral (C), and distal lateral (D). The mean values for equivalent von Misses stress for experimental testing were 14.26 ± 12.00 MPa (A), 11.68 ± 9.74 MPa (B), 6.14 ± 4.95 MPa (C), and 12.22 ± 9.81 MPa (D). From the micromotion contour plots in Figure 3, we found that the maximum value for micromotion was 4.76 *μ*m proximally and 13.03 *μ*m distally. This ensured bone ingrowth occurring in the bone—stem interface and fibrous tissue formation was prevented, which reflected the implant's fixation stability. The stress was normally distributed at metaphyseal region which was essential for primary stability fixation, preventing stress shielding at the proximal calcar as shown in Figure 4. The stresses demonstrated in FEA were 15–20 MPa (A), 20–35 MPa (B), 5–10 MPa (C), and 15–20 MPa (D). The safety factor for this new stem design was computed as 2.45.

**Figure 5:**
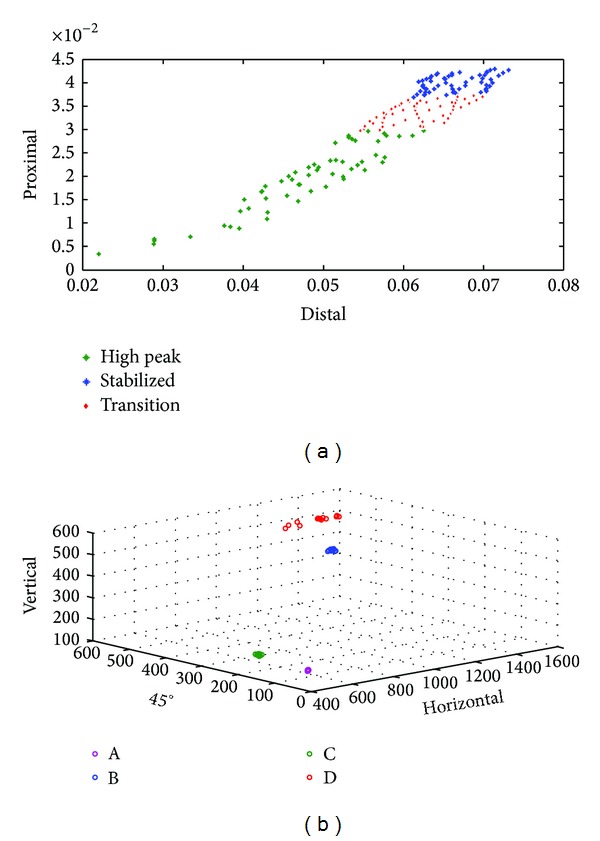
Pattern recognition from the vector support machine (a) micromotion and (b) strain.

We extracted active features using vector support machine classifier as shown in Figure 5. From this study, we managed to acquire 100% pattern recognition for both signals using SVM. Three micromotion classes (high peak, transitions, and stable) for proximal and distal LVDT and four strain classes (A, B, C, and D) were clearly discriminated in Figure 5. In Table 1, the RMS showed significant differences (*P* < 0.05) for comparisons against classes (high peak, transition, and stabilized) and between channels (proximal and distal). The RMS demonstrated *F* value of 669.79 (with *R* ^2^ = 0.66) between channels. In addition, the *F* values between channels for proximal region were shown as 339.92 (with *R* ^2^ = 0.80) and 151.17 for distal region (with *R* ^2^ = 0.64). Further analysis for multiple comparisons test between classes for RMS also denoted significant differences (*P* < 0.05). In Table 2, the RMS showed significant differences (*P* < 0.05) for both comparisons against classes (A, B, C, and D) and between channels (*ɛ*_1_, *ɛ*_2_, and *ɛ*_3_). However, comparison between classes medial (AB) and lateral (CD) was not statistically significant (*P* > 0.05). Advance analysis for multiple comparisons test between channels denoted a significant difference (*P* < 0.05). Furthermore, the RMS was statistically significant (*P* < 0.05) for multiple comparisons between classes, except between class A and C and class B and D which demonstrated almost similar mean value for these respective classes. On the other hand, the distribution between medial and lateral classes comparison demonstrated no difference between AB and CD.

## 4. Discussion

In general, the validation of the experimental testing correlated normally with the finite element analysis (FEA). Several studies have described the FEA as a fundamental preclinical testing tool with promising results [1–4]. The primary stability of the femoral stem during physiological loading and osseointegration are essential in determining the lifespan of the stem. Deficiency in fixation stability will cause thigh pain and loosening of the stem due to continuous disturbance during bone ingrowth [6]. Several studies proved that micromotion exceeding 150 *μ*m will cause fibrous tissue formation, while less than 40 *μ*m will stimulate osseointegration [6, 7]. Micromotion was found to be higher during the first load cycle compared to later cycles. The experimental and FEA results demonstrated abrupt changes between the first cycle and subsequent cycles. This occurred due to femoral stem fixation in the femoral canal that created prestress to the bone. Elastic micromotion from experimental testing showed that the femoral stem was stabilized around 10 to 11.5 *μ*m distally and 1.5 to 1.8 *μ*m proximally. On the other hand, micromotion from the FEA was 30 to 40 *μ*m distally and 20 to 30 *μ*m proximally. Although the experimental testing was well correlated with FEA, the result from FEA was slightly higher due to several limitations such as the friction coefficient (*μ* = 0.4) used in FEA for the implant—bone interface, simplified boundary conditions, loading configurations, and materials properties (inhomogeneous). Our study showed that the micromotion is within the adequate range which primarily promoted bone ingrowth at the implant—bone interface particularly at the metaphyseal region as illustrated in Figure 3. The optimal cross-section stem with the curvature radius tailored to the femora anatomical features conduced to primary stability and lower micromotion value.

On the other hand, another issue is the stress shielding due to bone atrophy at proximal calcar. Several factors influenced the load distribution in femoral stem which are implant geometry, implant, medullary canal interface orientation, and osseointegration [20]. Also, proximal bone ingrowth influenced stress distribution at the implant—bone interface maintaining bone stock in this region [22]. This present study showed that the maximum femoral stem stress did not exceed the yield strength of the bone, which was 160 MPa as shown in Figure 4 [23, 24]. A safety factor of 2.45 ascertained that the stem would not fracture the bone. The equivalent von Misses stress was computed to show the strain distribution at the triaxial rosettes. The strain results from experimental testing had patterns similar to the equivalent von Mises stress contour plot. The medial calcar (A and B), which generally experiences stress shielding due to adaptive remodeling, exhibited normal stress distribution in the FEA. This phenomenon can be described using Wolff's law which states that loads are transferred directly through femoral stem to the distal region bypassing the proximal region while performing hip replacement, which later caused bone atrophy to the medial calcar. Furthermore, the use of isoelastic femoral stems will cause stress shielding to the bone due to the stress to be reduced by half. However, the femoral stem in this study obtained excellent results both medially and laterally, which would prevent stress shielding from occurring and prolong the lifespan of the implant.

We reported several limitations in our finite element analysis. The femoral stem and medullary canal were assumed to be fully bonded without penetration. In addition, Pettersen et al. [1, 2] pointed out that the degree of contact pressure interference penetration during stem fixation is difficult to determine due to several factors such as stem size, femora size and quality, and force applied while performing surgery. In this study, our newly designed stem efficiently distributed stress proximally and presented micromotion under the threshold for osseointegration.

Pattern recognition of the primary stability of the cementless femoral stem is a new field of study which could determine the stable phase during the biomechanical testing. Although the FEA could predict the result of the implant, there are several limitations which influence this in silico method such as boundary and loading conditions, material properties, contact bodies, and mesh convergence. Any changes to these parameters will lead to different results which are not in compliance with the experimental results. In this study, we applied digital signal processing (DSP) to the raw signals for feature extraction and pattern recognition of the primary stability. We used root mean squares as the feature to feed the multiclass support vector machine (SVM) classifier for feature extraction and pattern recognition. The excellent result (100% pattern recognition) for the primary stability of the newly designed femoral stem proved that stem stability could be determined using this technique. The active features are clearly differentiated, which are also similarly applied to the strain as shown in Figure 5. This DSP method is easily applied, and it also saved computation time and showed presentable results. In addition, the result from DSP is in compliance with the FEA. This suggests that DSP could be used to determine the primary stability and could become an efficient preclinical tool for newly designed implants.

In micromotion study, three classes (high peak, transition, and stabilized) had been discriminated very well. This information was essential in determining the primary fixation stability of the femoral stem within the medullary canal. The deficiency in fixation stability will cause thigh pain and loosening of the stem due to continuous disturbance during bone ingrowth. This proposed method not only classified the classes with high accuracy but also provided the average value with its distribution around that region. For example, let us look upon the proximal region. The interface micromotion was distributed normally at the proximal channel with a mean value of 0.040 ± 0.002 for stabilized, 0.019 ± 0.007 for high peak, and 0.034 ± 0.002 for transition class. The channels (proximal and distal) were separated excellently (*P* < 0.001) with *F* value of 669.79 (*R* ^2^ = 0.66) and mean value of 11.821 ± 139.041 for proximal region and −3.373 ± 12.020 for distal region. Besides, the classes (high peak, transition, and stabilized) were distinguished very well with *P* < 0.001 with *F* value of 338.92 (*R* ^2^ = 0.80). The classification accuracy using SVM showed 100% which means that all classes for both channels were perfectly distinguished. This demonstrated that, within the adequate range as discussed above, the micromotion of the femoral stem promoted the osseointegration at the bone—implant interface for the proximal region which is in accordance with the experimental testing and FEA as shown in Figure 5(a).

On the other hand, the strain study focused on four classes (A, B, C, and D) which are located at medial and lateral region of the femur. Information regarding strain distribution is vital in ensuring that the force was transferred from proximal to distal region. A common problem after hip arthroplasty is stress shielding due to the differences in stiffness between the implant and femur. This phenomenon occurred at proximal calcar region which caused bone atrophy in the surrounding area and influenced the load transfer pattern to the femur. To comprehend more about this method, for instance, let us take class A. The strain was normally distributed at the medial calcar at proximal region with each channel (*ɛ*_1_, *ɛ*_2_, and *ɛ*_3_) and was statistically significant (*P* < 0.001) with *F* value of 340 622 (*R* ^2^ = 0.99). The mean values denoted for each channel based on the three orientations of the triaxial rosettes are as follows: *ɛ*_1_ was 455.310  ±  2.457, *ɛ*_2_ was 40.135  ±  0.509, and *ɛ*_3_ was 172.878  ±  1.791. *ɛ*_1_ represented the horizontal axes (0°); *ɛ*_2_ represented the 45°, and *ɛ*_3_ represented vertical axes (90°) as shown in Figure 1(d). This information demonstrated the strain transferred with its distribution according to these three orientations at region A. Furthermore, all classes (A, B, C, and D) were statistically significant (*P* < 0.001) with *F* value of 66.36 (*R* ^2^ = 0.41). However, the medial (AB) and lateral (CD) located rosettes were not statistically significant (*P* < 0.16) with *F* value of 1.98 (*R* ^2^ = 0.007). This was illustrated in Figure 5(b) which showed that the medial rosettes (A and C) were located at similar axes in horizontal (*ɛ*_1_) and 45° (*ɛ*_2_) axes with the mean value stated above. However, features A and C were confirmed as not statistically significant (*P* = 0.6304) from the multiple comparisons test. The classification accuracy using SVM showed 100% which means that all classes for both channels were perfectly discriminated. This demonstrated that, within the sufficient range as discussed above, the micromotion of the femoral stem distributed the strain at these locations similar to the experimental testing and FEA.

There are several limitations in our study. Firstly, the measurements taken in the experimental testing were restricted to four triaxial rosettes for strain distribution at the metaphyseal region and two LVDT for micromotion at the proximal and distal region. The load transferred through femoral stem normally caused the stress shielding at the medial calcar which resulted in bone atrophy and loosening. In this study, the strain was normally distributed at the metaphyseal region due to optimal contact area of this newly designed femoral stem. The micromotion also demonstrated less than 40 *μ*m which promoted osseointegration between stems—bone interface for both proximal and distal regions. Secondly, only one stem type was used for experimental testing which suited the Asian femur anatomical features. More commercial off-the-shelf femoral stem types were required for experimental testing in the future which contributed more samples data to acquire more reliable DSP method for primary stability recognition. Finally, only RMS was used as the time domain feature with the SVM as the classifier. Further study using different time domain features such as maximum absolute value (MAV), kurtosis (KUR), mean value (MV), waveform length (WL), and simple square integral (SSI) could be tested so that the requirement for training can achieve the reliable system. Furthermore, the experimental results provided evidence of the possibility of selecting the best feature values in order to improve the robustness of the DSP model.

## 5. Conclusion

We would like to stress the application of digital signal processing (DSP) method in determining the femoral stem primary stability with high pattern recognition accuracy in biomechanical testing. Despite the practical constraints involved, significant results have been obtained through the DSP system which validated the experimental results with good correlation which could be applied as preclinical tools. Nevertheless, further study regarding different femoral types and time domain features and classifiers were required to develop a reliable DSP method. However, this method demonstrated excellent result in discriminating each class in strain and micromotion with lower computational cost and less preset parameters.

## Figures and Tables

**Table 1:** Analysis of micromotion variance for comparison between channels and classes.

	*N*	*F* value	Pr > *F*	*R*-square
Between channels	342	669.79	<0.0001	0.663297
Between classes				
Proximal	171	338.92	<0.0001	0.801379
Distal	171	151.17	<0.0001	0.642819

**Table 2:** Analysis of strain variance for comparison between channels and classes.

	*N*	*F* value	Pr > *F*	*R*-square
Between channels				
A	72	340622	<0.0001	0.999899
B	72	180487	<0.0001	0.999809
C	72	37744.3	<0.0001	0.999087
D	72	2278.67	<0.0001	0.985085
Between classes				
A versus B versus C versus D	288	66.36	<0.0001	0.412109
AB versus CD	288	1.98	0.1603	0.006881
